# The Assertive Resolution of Conflicts in School With a Gamified Emotion Education Program

**DOI:** 10.3389/fpsyg.2018.02353

**Published:** 2018-11-30

**Authors:** Gemma Filella, Agnès Ros-Morente, Xavier Oriol, Jaume March-Llanes

**Affiliations:** ^1^Faculty of Education, Psychology and Social Work, University of Lleida, Lleida, Spain; ^2^Facultad de Educación y Ciencias Sociales, Universidad Andres Bello, Santiago, Chile

**Keywords:** emotion regulation, gamified program, coexistence, conflicts, adolescence

## Abstract

Coexistence in schools inevitably carries a higher risk of conflicts among peers. This fact can be detrimental to the well-being and academic achievement of the students. In many developed countries, about 90% of the pupils in compulsory secondary education report witnessing assaults among peers. In this regard, recognizing, controlling and managing emotions is key to ensure a healthy and effective interaction with others. Negative emotions, such as anger, can trigger conflicts or even episodes of violence if not regulated properly. Interactive tools, such as specialized software’s, have shown high rates of efficacy for the training of different kinds of competences like the regulation of emotions. The aim of the present work is to describe the *Happy Emotional Education Program and* its effects over a sample of secondary school students. This software focuses in the training of emotional competences of the students with the objective of solving conflicts in a more assertive way. The design employed in the present work was a quasiexperimental design with pretest and posttest with a control group. Results show that *Happy Emotional Education Program* contributes to the management of emotions and the absence of negative affect or anxiety in a significant way. Additionally, the constant use of this interactive tool enhances motivation and the learning process of students. Results also indicate the importance of assessing the effects of the program in the long term. This would enable researchers to further assess the effects of the program over those emotional competences that are more resistant to change given its stable nature.

## Introduction

The improvement of coexistence and the prevention of bullying and aggression in schools have become subjects of high interest in the international arena. The attention that these topics have received during the latest decades responds to the need to face the high rates of violent episodes and aggression in our scholar centers. For example, latest reports show that one out of four children has experimented bullying in school (Gabarda, 2014; Save the Children, 2015) and that up to a 45% of the students can be identified as victims of a violent situation in their school environment (Kasen et al., 2004; Dinkes et al., 2009).

The scientific community has emphasized that this kind of phenomena inevitably involve negative consequences for the general well-being and affective states of the students, simultaneously hindering and challenging a correct and healthy learning process. Wolke et al. (2012) for instance, proved in 2012 with a study with 6050 mothers and their children that those individuals who had received intentional harm inflicted by peers also showed precursors or markers on the trajectory toward the development of symptomatology of Borderline Personality Disorder in childhood. Additionally, evidence shows that when the classroom climate and, by extension, coexistence among peers, is negative, the scholar center becomes more vulnerable to situations of bullying (Wang et al., 2014). Thus, results of the latest evidence unquestionably state that suffering abusive situations by peers has a detrimental effect in the academic results of the pupils, as well as the personal development of the victim, who becomes highly prone to an unhealthy state of mind and to the perception of the center as threatening and violent (Cerezo, 2008; Martorell et al., 2009; Caballo et al., 2011; Pérez-Fuentes et al., 2011). In the opposite manner, when coexistence in school is adequate and positive, there is a clear improvement in the participation and motivation of the students, and ultimately, in the learning process. This is reinforced by an adequate self-esteem of the students, prosocial skills and a healthy environment (Álvarez-García et al., 2013).

Related to the relationship among peers and its grounds, it is well established among the scientific community that many of the disruptive behaviors or aggressions that occur among peers are not due to an excess of hostility. Rather than being a problem of violence *per se*, they can better be attributed to a lack of skills and strategies to manage emotions and solve the social problems in an effective way (Ortega, 2010). This can be given a positive reading, since a number of studies show that although a poor management of emotions can bring to conflict situations, aggressions and intimidation, a systematic training of these skills can facilitate an important decrease in this kind of behaviors (Extremera and Fernández-Berrocal, 2003; Pérez-Escoda et al., 2013; Romera et al., 2015; Filella et al., 2016; among others).

Evidence so far widely recognizes that all the emotional responses are necessary and useful (Öhman and Mineka, 2001). However, when emotions are mismatched to our needs and become disrupted, it is of the foremost importance to use strategies in order to regulate our emotional state (Gross and John, 2003; Aldao et al., 2010). Empirical evidence shows that an effective management of emotions leads to several important outcomes, such as mental health (Gross and Muñoz, 1995), subjective and psychological wellbeing (Balzarotti et al., 2016), and relationship satisfaction (Murray, 2005). On the contrary, emotion dysregulation may lead to certain forms of psychopathology (Aldao et al., 2010) and maladaptive behaviors (Harrison et al., 2010). Studies in this direction, thus, show the need for action and the urge to develop efficient training programs. This would help improving emotional competencies in scholar centers, at the same time that there is an enhancement of the positive climate and a prevention of aggressive manifestations among peers (Extremera and Fernández-Berrocal, 2003; Ortega, 2006; Pena and Repetto, 2008; Pérez-Escoda et al., 2012; Miñaca-Laprida et al., 2013; Blair and Raver, 2015).

The design and implementation of programs that train certain socioemotional skills is not altogether new. Researchers and professionals have sought to find adequate socioemotional programs that improve the learning process of the students for over a decade (for example, Pascual and Cuadrado, 2001; Filella et al., 2002; Güell and Muñoz, 2003; Renom, 2003; López-Cassà et al., 2005; Greenberg and Kusché, 2006). One of the pioneers in the approach of social and emotional skills was the Collaborative for Academic, Social and Emotional Learning (CASEL), which started applying programs in the North American schools on the premise that those problems that have an impact during childhood and adolescence in schools are mainly explained by difficulties in the social and emotional field. Thus, these programs have the final objective of fostering these kind of skills since a very early age, promoting a positive and motivating environment for the child through novel methodologies (Greenberg et al., 2003).

Benefits stemming from this type of interventions in emotional education have rapidly spread in the scientific field, proving that there have been significant improvements in the prosocial behavior and an important decrease of negative and disruptive behaviors (Eisenberg et al., 2004; Pérez-González, 2008). These results are also consistent with previous studies carried out in the neuroeducational field (Davidson, 2012), which show that the benefits obtained by well-designed socioemotional programs go beyond momentary changes and that they bring long-term beneficial effects at a biological, psychological and social levels.

Ultimately, the development of socioemotional skills in the scholar context has proved to be an important enhancer of a healthy development of any human being. The promotion of a stable self-esteem, the fostering of motivation for learning, and the promotion of mental health are just a few examples that can be explained by a solid and efficient set of socioemotional competences (Jiménez, 2009). Additionally, those interventions that enhance the management of negative emotional states have also proved to increase academic achievement in children in the short and also in the long-term (Extremera and Fernández-Berrocal, 2003, 2004; Pérez-Escoda et al., 2012, 2014).

In view of these findings, we can state that there is a need to train and enhance emotion education in school. To achieve this objective, researchers need to seek and implement novel methodologies that ensure the improvement and the engagement from the educative community. In this sense, authors and professionals have started developing and exploring new lines of intervention and programs, such as virtual platforms. Among the virtual mediated experiences, videogames are becoming increasingly popular and they are attracting the interest of researchers about their opportunities for positive individual functioning, increased motivation and learning enhancers in schools (e.g., Gaggioli et al., 2014; Villani et al., 2018). For this reason, in the present work we aim to show the evaluation of an educative gamified program underpinned in the model of emotional competences developed by the Group of Psychopedagogical Orientation (Bisquerra Alzina and Pérez Escoda, 2007) which focuses in five dimensions: emotional awareness, emotional regulation, autonomy, social competences, and life’s competences (Bisquerra Alzina and Pérez Escoda, 2007).

Happy software’s. Happy 12–16.

Happy 12–16 is a gamified program which is part of the Happy software’s. Happy 12–16 is specifically designed to help adolescents aged from 12 to 16 improving the management of their emotions by training their emotional competencies. As mentioned above, evidence has proved that those individuals that display a successful management of emotions can give a better answer to those conflicts that they encounter during their daily life (Webster-Stratton and Reid, 2004).

This program is structured in 25 conflicts, 15 of which take place in the scholar context and the other 10 take place among siblings in the household or family context. Three examples of the conflicts that students can encounter in the software are the following:

In the scholar context: “*You stain with chalk your classmate of sub-Saharan origin while you insult him telling him he looks better like this*,” or *“You observe how your classmates have erased Robert from the Whatsapp group of the class because the group administrator does not like him.”*

In the family context: “*Your brother needs to focus in order to study for an exam but you put on very loud music.”*

The student will have to choose among different answer possibilities (assertive answer, passive answer, or aggressive answer) for each conflict. However, only the assertive response is considered correct. Every time the student chooses the assertive answer, he will receive bonus points.

It is important to note that the student does not always have the same role when playing the videogame. The participant can be the bully, the victim or the observer or bystander. This helps promoting the number of possibilities and strategies that the students will have to display and learn in order to select a proper answer.

Happy software’s stem from the Psychopedagogical Orientation Group (GROP) theory of emotional competences. The GROP takes into account the orientations of the developmental psychology, cognitive and linguistic skills and the development of the six moral stages developed by Piaget-Kholberg to build the foundation of the gamified programs. Also, the strategies of emotion regulation used in the programs draw from the model of Gross (2007), which points out three strategies of regulation that are applied in the Happy software’s: attention deployment, reappraisal and situation selection.

The resolution of the conflict situations follows the scheme described in the following figure:

Taking into account all this information, the main aim of the present work is describing the intervention and the results of the program Happy 12–16 in a group of students in Spain. Specifically, we intend to assess the effects of the Happy 12–16 software on the emotional competencies and other important correlates, such as the levels of anxiety of students, the climate in the classroom and the academic achievement of the students.

## Materials and Methods

### Participants

The sample of the present study was composed of a group of students of secondary education schools in the regions of Lleida and Huesca (Spain). Among the participant schools, there were those schools which underwent the experimental condition and other schools which constituted the control group.

The final sample consisted of a total number of 903 students. 471 of them were males (52.2%) while 432 (47.8%) were females. All the students of this group were in 1st (*n* = 440; 48.7%) and 2nd (*n* = 463; 51.3%) grades of compulsory secondary education. The average age of the students was 12.63, with a standard deviation of 0.608.

The experimental group included 472 students of 7 different high-schools (52.3% of the total) and the control group was constituted by 431 students of 4 voluntary centers (47.7% of the total). In the analyses, no statistically significant differences among groups were found.

### Instruments

The instruments to tackle the variables included in the study were:

*Emotional Development Questionnaire for secondary school* (QDE SEC; Bisquerra Alzina and Pérez Escoda, 2007). This instrument has two versions, one for elementary school students and another for adolescents. In the version for adolescents, there are 35 items. It is possible to obtain a global score or a score for each one of the subscales described by the Group of Psychopedagogical Orientation (GROP): Emotional Awareness, Emotion Regulation, Emotional Autonomy, Social Competences and Life’s Competences (Bisquerra Alzina and Pérez Escoda, 2007). In the present work, we used the global score for being more informative regarding the objectives of the study and showing a higher internal consistency. In fact, when psychometrically analyzed, the instrument showed an adequate internal consistency, with an Alpha coefficient of 0.83 for the total score, and 0.70–0.80 for the different subscales.

*Stait-Trait Anxiety Inventory* (STAI; Spielberger, 1973; Seisdedos, 1982). This instrument was created by Spielberger (1973). Later, it was adapted to the Spanish population by Seisdedos (1982) and it kept its original two scales with 20 items each that assess Anxiety-State (A-E) and Anxiety-Treat (A-T). In this case, the instrument showed an excellent internal consistency with an Alpha of Cronbach of 0.93. For the purposes of the present work, only the A-E scale (Alpha coefficient of 0.94) was administered, since the main interest regarding anxiety was to explore the anxiety levels of the individuals in the moment of the study.

*Happy 12–16 gamified software*, described in the previous section, was administered to students of the experimental group during their weekly hours of tutoring (2 h per week), as part as their curricula. Administration of Happy software was always under the supervision of a trained teacher who guided and supervised the sessions.

Finally, *academic performance* was evaluated with the average marks of all the subjects: Biology and Geology, Geography and History, Spanish Literature, English Literature, Physical Education, Ethics, and three different subjects that students could choose during the academic course. The gathering of data regarding academic performance was carried out in December (first evaluation of the academic year) and in June (third and last evaluation of the academic year).

### Procedure

Before the study took place and in order to ensure that the project would be implemented in an optimal way, the research team contacted the Department of Education in Spain. The present research was introduced to all the professionals of the Department in different meetings. Also, the research plan was studied in detail by the Department of Education and the management teams of each scholar center, achieving the consent of the Government’s Department of Education.

It is also important to note that this study was exempt of ethics approval beyond the approval of ethics committee of the University of Lleida itself, since it was considered non-invasive and school related. However, given the young age of the students, both parents and students were thoroughly informed about the research. Parents were informed in the first meeting of the academic course with the management team. Those who did not want to participate from the beginning were given the option to leave. Those who showed interest had the opportunity to ask investigators and teachers and only those who were 100% sure of their participation were included in the study. Parents gave oral consent to the schools to enable their children to undergo the study. Once the students filled out the questionnaires, they also gave consent of their participation. This consent procedure was approved by the Committee that approved the study itself.

Thereupon, there was a first contact with those schools that showed their willingness to participate with the objective of explaining them the research project associated with the existent software’s (Happy 8–12 and Happy 12–16), and most specifically Happy 12–16. Given the novelty of the software’s and the interest in an extensive analysis of its effects, two researchers of the team oriented, guided and trained the teachers and management teams of each one of the experimental schools participating for the administration of the software’s. The training on Happy 12–16 took over 30 h distributed in sessions of 1 h. Only after completing the training, the posttest protocol was administered. Subsequently, those centers of the experimental condition followed the 12–16 Happy training program and, finally, all the remaining data of the students was collected with the posttest protocol. The design of the present research was a quasi-experimental design with pretest and posttest and a control group.

### Data Analysis

In order to assess the effect of the Happy 12–16 software in the experimental group a General Linear Model (GLM) for repeated measures was carried out for each subscale and for the total QDE SEC, STAI-E and Academic achievement. Group was the between factor, and phase (Pre vs. Post) the within factor. *P*-values were adjusted according to the stepwise Holm procedure to correct the familywise error rate with correlated scores (Bender and Lange, 2001). Data was processed using the SPSS 20.0 software package.

## Results

Descriptive data of the quantitative variables with the analysis of the comparison pretest and posttest of both subsamples of the study can be seen in Table 1. Descriptive statistics of the demographic variables were obtained in the very beginning of the study for both groups (control and experimental) in order to analyze and compare the homogeneity of the sample.

**Table 1:** Average values of the results of the tests for the control (*n* = 432) and experimental (*n* = 472) groups of secondary school students.

Test	Group	Pre Happy 8–12 score	Post Happy 8–12 score	Average change pre/post	Average change F; *p*-value^∗^	Effect size (d)
QDE SEC total	Experimental	6.09	6.05	0.04	3.77; 0.52	0.13
	Control	6.26	6.18	0.08		
QDE SEC emotional awareness	Experimental	7.38	7.57	0.19	7.71;.04	0.19
	Control	7.24	7.32	0.07		
QDE SEC emotion regulation	Experimental	5.12	5.06	0.06	0.17; 0.68	0.03
	Control	5.22	5.25	0.03		
QDE SEC autonomy	Experimental	5.82	6.01	0.19	6.02; 0.03	0.16
	Control	5.96	6.02	0.06		
QDE SEC social competence	Experimental	5.96	5.97	0.01	0.20; 0.65	0.03
	Control	6.13	6.03	0.10		
QDE SEC life’s competences	Experimental	6.30	6.54	0.24	26.25; 0.005	0.34
	Control	6.50	6.77	0.17		
STAI-E	Experimental	43.71	44.70	0.99	5.02; 0.02	0.15
	Control	42.67	43.22	0.55		
Academic achievement	Experimental	5.89	6.07	0.18	33.08; < 0.001	0.38
	Control	5.87	5.98	0.11		

*^∗^Adjusted by Holm procedure.*

At the time of data collection, students completed QDE-SEC for the global scale and for its subscales. As Table 1 shows, these results show that QDE-SEC scores of those students who underwent the intervention with Happy software in the experimental group significantly improved. The students composing the control group did not show that same improvement in their scores. However, it is important to strike the fact that the global score of QDE-SEC, although it showed a great tendency, it did not become statistically significant. Additionally, the effect size was smaller than 0.15 (*d* = 0.13), suggesting that there may not be any effect (Hattie, 2009). For this reason, it was highly important to study in an individual fashion each one of the subscales of the instrument. In this second part of the analysis, as it is shown in Table 1, three scales showed a statistically significant difference among the experimental and the control group after the implementation of the program: emotional awareness [*F*(7.71); *p* < 0.01], emotional autonomy [*F*(6.02); *p* < 0.01] and life’s competences [*F*(26.25); *p* < 0.001]. However, size effects remained small ranging from 0.17 to 0.34).

Regarding social competences and emotion regulation subscales, no statistically significant changes were observed, although a small effect size was observed (see Table 1), which may explain the fact that the global scale did not result statistically significant.

As it was explained above, anxiety was also a relevant variable measured in the study for all students. The outcome obtained shows that there is a statistically significant increase in the levels of anxiety of those students that composed the experimental group [*F*(5.02); *p* = 0.002; *d* = 0.15], which is an unexpected result which may require an adjustment in the author’s approach.

Differently, academic results showed an important and statistically significant increase as well as a medium size effect in the experimental group after the students benefited from the training program with Happy 12–16. This important improvement was not observed in the control group.

## Discussion

Contrary to popular belief, evidence shows that those conflict situations that arise in the school context are closely related to the ability of the students to manage and control their emotions (e.g., Webster-Stratton and Reid, 2004). That is, those students who have more difficulties regulating their emotional states and being assertive will also show a greater involvement in conflicts (Bisquerra, 2014; Filella, 2014). Also, a poor management of emotions and social skills has also been linked to a worsened academic achievement. Additionally, difficulties in regulating emotions can increase the vulnerability of the individual to develop symptoms of pathology or even full-blown anxiety or depression (Extremera and Fernández-Berrocal, 2004; Spinrad et al., 2004; Wolke et al., 2012).

For the last several years, there has been an important rise of programs specifically designed to improve social and emotional skills. This fact has greatly helped the development of new interventions and methods that have proved to be every time more promising for children and adolescents, both at the academic field and at the personal and social scopes (Greenberg et al., 2003).

The goal of this present work was to explore the effects that a gamified program of social-emotional competencies may have among adolescents aged 12–16. The implementation of this method comes after several studies pointing out that the motivation of the students, who are very connected with interactive formats and technological advances, significantly increased when this type of programs are applied (for example, Deterding, 2012). At the same time, higher motivation levels improve the learning processes of the students and help teachers to carry out their professional tasks with greater ease (e.g., McGonigal, 2011; Gaggioli et al., 2014).

The results of the present research show that, as it was expected, those individuals that received training with the gamified program showed a higher and significant tendency to improve their emotional competencies. Differently, the control group did not show important changes in their levels of emotional competencies. However, it is important to note that the effect of the learning process in emotional skills was modest and that effect sizes were small for all the scales except of anxiety. This can be explained by the fact that variables such as the emotional competencies, which involve a certain amount of stability, require a long time to its total change and training.

**FIGURE 1:**
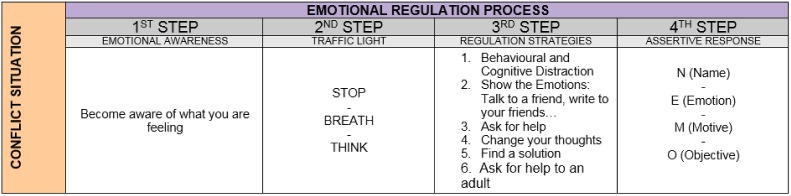
Steps followed in the Happy software’s following the emotional regulation process (GROP). All participants have to follow this steps when facing a conflict during the game. If the process is successfully completed, the participant will get a high score. Contrary to that, if the participant obtains a low score, the need of training the process will arise.

It is also important to emphasize that there was a more clear and significant effect for emotional awareness than for other competencies. Far from being surprising, this effect is only understandable since it unquestionably constitutes a reflection of the natural acquisition of the emotional competences and its training, which first requires or becoming aware of one’s emotions (see Figure 1; Gross and John, 2003).

Regarding the regulating process, our results showed a slower and more difficult pace for the acquisition of this particular skill, which can be explained by the traditional cognitive view that points out the difficulty of transformation in those beliefs or emotional processes that are very substantial in our core of thoughts (Beck et al., 1979). Despite this fact, results showed a high percentage of improvement in the *post hoc* questionnaire of CDE-SEC.

In a complementary manner, life competencies and autonomy also increased in a significant way in the experimental group and showed slightly higher effect sizes. These competencies, related to variables such as self-esteem and the capacity of the individual to independently managing oneself among different facets of life, responded in the expected way to the implemented program. As explained in the case of emotional awareness, these competencies are crucial in the development and improvement of the other skills, which are more complex and have a more stable nature, such as emotion regulation or social competencies (Spear and Kulbok, 2004).

On the other hand, the levels of anxiety-state that the students presented showed a tendency to grow in both groups. This fact, although not expected, is coincident with the previous evidence that indicates that anxiety can be a crucial factor during the development period of adolescence and that it can be attributed to this phase of the vital development (Boyd et al., 2000;Öhman and Mineka, 2001; Neil and Christensen, 2009). Additionally, recent data indicates that anxiety disorders are among the most commonly experienced and diagnosed conditions of childhood and early adolescence (Grills-Taquechel and Ollendick, 2012). Given this circumstance, it is important to take into account that, although there are different ways to appraise emotions among individuals, there is also an undeniable tight connection between emotion regulation and anxiety (e.g., Amstadter, 2008). Thus, there is a critical need to prevent the anxiety that students may feel since childhood by the development and enhancement of a proper management of emotional competences when possible (Davidson, 2012; Pérez-Escoda et al., 2014).

In a different manner, among the variables of academic achievement, the tendency of improvement was significantly different in the experimental group and effect size was medium. Although previous studies point out the importance of social and emotional levels for the improvement of academic achievement, especially when mediated by variables such as engagement (for example, Dotterer and Lowe, 2011), these results are probably explained by the fact that the marks were gathered right after the training with the software at the end of the academic course. In this sense, a reassessment in the long term could yield more detailed results.

In future studies, the possibility of an assessment or a follow-up in the long term would be highly beneficial, bringing the possibility to study the durability of the effects of the training. Moreover, we believe that effect sizes can improve if intervention continues for a longer period of time. Due to the stable nature of certain variables, such as the competencies of emotion regulation or anxiety trait, which can have an effect in anxiety-state, research that embraces more than one academic course could possibly bring substantial evidence regarding the changes in the long term. In addition, future investigations should consider using other kind of measures that complement the subjective vision of psychometric assessment, such as social measures or even biological markers.

## Author Contributions

GF designed, created, and participated in the process and creation of the study and the manuscript. AR-M was involved in the writing and creation of the manuscript. XO and JM-L contributed to the revision of the methodological aspects and helped in the creation of the article.

## Conflict of Interest Statement

The authors declare that the research was conducted in the absence of any commercial or financial relationships that could be construed as a potential conflict of interest.
